# Salivary parotid cyst as an occurred complication of Subangulomandibular approach of mandibular subcondylar fracture: A case report

**DOI:** 10.1016/j.amsu.2020.11.078

**Published:** 2020-12-01

**Authors:** Rachid Aloua, Faiçal Slimani

**Affiliations:** a Faculty of Medicine and Pharmacy, Hassan II University of Casablanca, B.P, 5696, Casablanca, Morocco; b Oral and Maxillofacial Surgery Department, CHU Ibn Rochd, B.P, 2698, Casablanca, Morocco

**Keywords:** Sialocele, Parotid gland, Salivary duct, Mandible, Condylar fracture

## Abstract

**Introduction:**

A number of papers exist about post-traumatic silaocele. Iatrogenic sialocele has been described as complication of parotid surgery but there is no specific data about the cases of sialocele and salivary duct surgical injury in condylar fracture management.

**Presentation of case:**

A 36-year-old man was reported to the maxillofacial emergency department for mandibular sub-condylar fracture and internal fixation of the sub-condylar fracture was planned. Postoperatively he presented a salivary cyst which was managed by conservative treatment.

**Discussion:**

Iatrogenic injury to the salivary duct and sialocele formation observed in the post-operative period of surgical treatment of a condylar fracture by sub-angulomandibular approach. Maxillofacial surgeons should be aware of this complication and the importance of adopting a conservative treatment whenever it is possible. Botulinum toxin injections can be performed as a first-line treatment for these complications.

**Conclusion:**

A conservative management was adopted by pressure dressings and percutaneous needle aspiration of the swelling, which was useful to absorb cystic content and helped healing.

## Highlights

• No specific data about the cases of sialocele and salivary duct surgical injury in condylar fracture management.
• The iatrogenic lesion of the salivary gland is rarely observed in trauma surgery when the subcondylar fracture is revealed.
• The principle of treatment is to dry up salivary secretion.
• Maxillofacial surgeons should be aware of this complication and the importance of adopting a conservative treatment whenever it is possible.

## 1 Introduction

Mandibular condylar and sub-condylar fractures are common in facial trauma [2]. The iatrogenic complications of the open treatment include pain, restricted mandibular movement, muscle spasm and deviation of the mandible, malocclusion, and pathological changes in the TMJ, osteonecrosis, facial asymmetry, and ankylosis, irrespective of whether treatment was performed or not [1,2].

## 2 Case report

A 36 years-old man with no medical history was reported to the maxillofacial emergency department following severe facial trauma as a result of an automobile accident. He complained of pain in his jaw and was unable to open his mouth.

Initial physical examination showed abrasions and lacerations on the facial skin and the lips. Limited opening of the mouth and lateral deviation of the mandible toward the right side on mouth opening was noted. Intraoral examination revealed no abnormalities.

The Initial panoramic radiography revealed a displacement mandibular sub-condylar fracture with a decreased vertical height of the ramus in the fractured side.

**Fig. 1:**
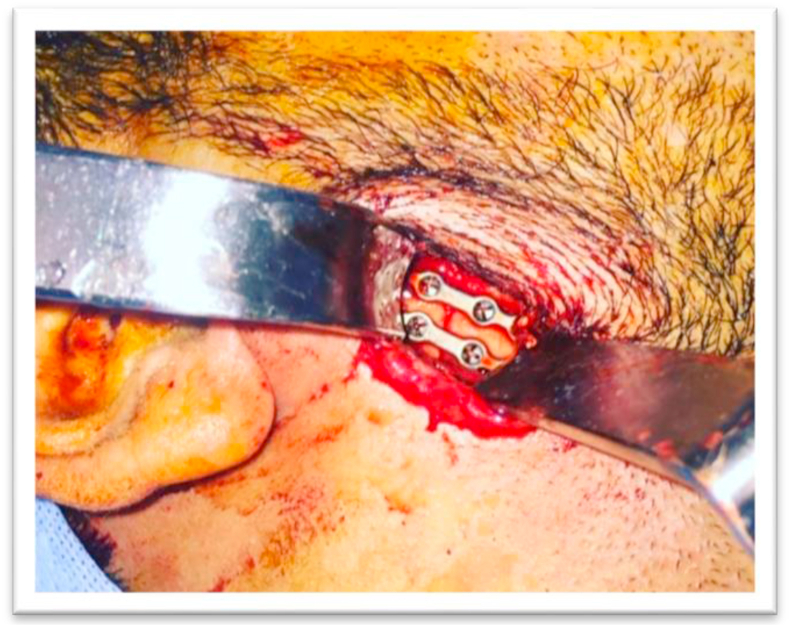
Sub-angulomandibular approach.

Based on the positive medical history and clinical examination, open reduction, and internal fixation of the sub-condylar fracture were planned. General anesthesia was administered through nasotracheal intubation. The sub-angulomandibular approach was used for the exposure of the fracture site Fig. 1. Surgical intervention was performed by our chief resident who has 5 years of operative experience. Perioperatively after the incision of the platysma, and the detachment of the plane under platysmal, we noted the presence of parotid tissue at the level of our fracture. The postoperative period was uneventful with no complications such as facial nerve palsy.

**Fig. 2:**
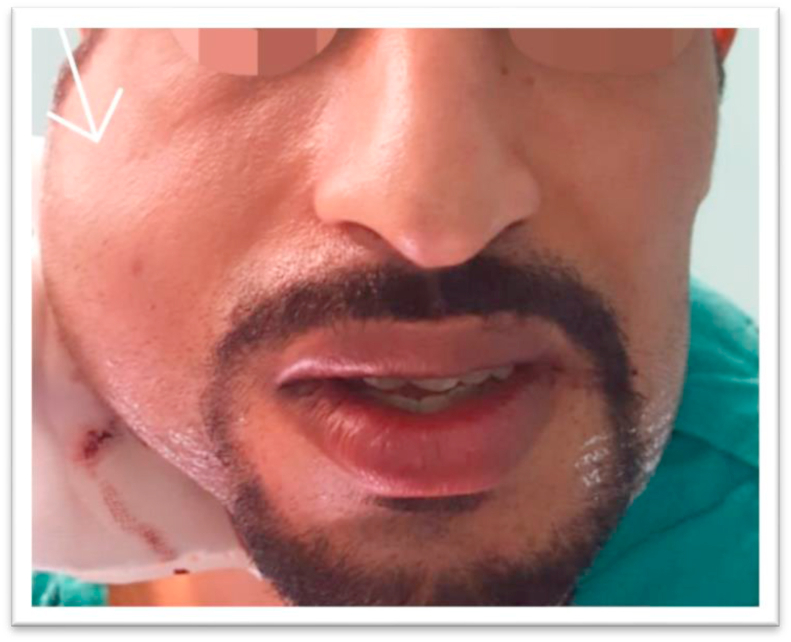
Facial view illustrating fluctuating mass on patent's right check.

On the third day of the postoperative period a fluctuating mass measured about 6 cm in length and 5 cm in width in the right parotid region appeared (Fig. 2). There was no associated pain or alteration of facial function as well as no motor or sensory deficits were observed.

On bimanual palpation an ill-defined and resilient mass was noticed, the percutaneous puncture of the mass was performed obtaining 20cl of yellow liquid.

At the 30-day follow-up visit, complete recovery was noticed, and the residual swelling had totally disappeared after multiple aspirations and compression dressings. Routine follow up 3, 6 and 12 months later showed no signs of recurrence.

This case has been reported in line with the SCARE criteria [9].

## 3 Discussion

Sialocele is an inflammatory pseudo-capsule that contains salivary secretions within the soft tissues of the cheek, without proper drainage [3]. Clinically, it presents as a painful, tight but not hard swelling of the face. These complications are the result of the lytic action of components in the saliva that prevent healing with suture release.

This is the complication especially of neglected parotid pleasures, without surgical exploration, the average time to their onset is seven days for fistula and 12 days for sialocele.

In facial trauma, the sub-angulomandibular approach, which we used for our patient, the literature reports a large number of cases of facial nerve damage (up to 37% of operations in certain series [[4], [5], [6]]), usually transient and limited to the marginal and buccal branches. They can be related to a direct trauma of these branches during the sub-platysmal dissection or the section of the masseter. Salivary complications are mainly reported for the *trans*-parotid approach and preauricular to the mandibular condyle. In our case, the salivary involvement can be explained by the involvement of the masseteric prolongation of the parotid artery which is demonstrated during the surgical intervention.

The principle of treatment for this complication is to dry up salivary secretion to allow healing [[3], [4], [5]]. Preserving the function of the parotid is no longer the primary objective. Surgical management of complications from wounds of the parotid gland and its direct or surgical channel seems to be less and less retained because of its morbidity, poor results, and especially the approval of conservative treatment [4]. Different methods of surgical treatment were used: ligation of the canal, section of the auriculotemporal nerve or Jacobson's nerve, parotidectomy.

A variety of treatments have been proposed for parotid sialoceles [8]. These include multiple aspirations and compression dressings; late primary repair or reconstruction of the duct; creation of a controlled internal fistula; superficial or total parotidectomy; parasympathetic denervation (sectioning of the auriculotemporal nerve); anticholinergic substances such as probanthine or atropine; local radiation therapy and ductal ligation [7]. Most of these procedures are invasive with risks of injury of the facial nerve, with variable and often poor success rates [4,6]. Currently, botulinum toxin injections can be performed as a first-line treatment for these complications. In our case, we adopted a conservative treatment by compressive dressings and percutaneous puncture, with good clinical results.

## 4 Conclusion

The iatrogenic lesion of the salivary gland or the salivary duct is rarely observed in trauma surgery when the sub-condylar fracture is revealed. If not treated duct injury may result in such complications as sialocele or ductal cysts. Numerous techniques for the management of salivary duct injuries have been proposed, but botulinum toxin represents an excellent alternative for the treatment of sialoceles and salivary fistulas because of its effectiveness, its rare side effects and its low binding nature.

Maxillofacial surgeons should be aware of this complication and the importance of adopting a conservative treatment whenever it is possible.

## 5 Provenance and peer review

Not commissioned, externally peer reviewed

## Appendix A Supplementary data

Application Application
